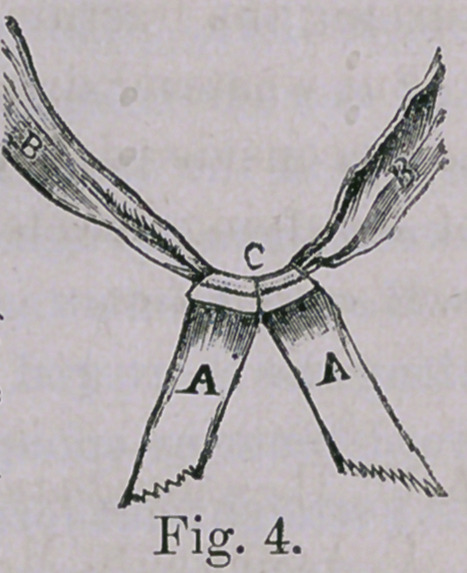# Hints on the Treatment of Fractures of the Lower Extremeties

**Published:** 1874-01

**Authors:** J. F. Woods

**Affiliations:** Toledo, Ohio


BUFFALO

### Medical and Surgical Journal.

VOL XIII. JANUARY, 1874. No. 6.

### Original Communications.

:o:

ART. I.—*Hints on the Treatment of Fractures of the Lower Extremities.* By J. F. Woods, M. D., Toledo, Ohio.

In a case of fracture we have for consideration the form and extent of injury of the bone and greater, or less bruise and laceration of the adjacent soft tissues.

Whatever the extent and kind of injury may be, the process of repair is essentially the same and the principles of treatment correspondingly uniform.

The method adopted should of course be such as to afford the greatest possible facility for these delicate processes, and in ordinary cases may be summarized as follows:

First—The *early* adjustment of the fractured bone in nearly as possible a proper position.

Second—The retention of the fragments in that position without being moved—thus avoiding interference with the efforts of nature.

Third—The treatment of any inflammatory or other complications that may arise.

It seems to me that there can be no reasonable doubt as to the duty of *immediate adjustment,* or at most within the period during which shock maintains a comparative relaxation of the muscles. At this time all the necessary examinations and adjustments may be effected with comparatively little pain—a matter of great consequence; nothing can be gained by delay. The unnecessarily *rude handling* that is so often practiced by incompetent or pretentious surgeons cannot be too strongly condemned, and the "pulling the bones into place" may be effected almost without pain and within a short time, by slight but *continuous* extension, the lateral force of the stretched muscles aiding in aligning the fragments, so that often little or no other lateral pressure is needed.

The keeping of the fractured extremities *absolutely quiet* from the first is not fully appreciated. Any movement of the spiculated extremities cannot, in my judgment, fail to do some harm, and I see nothing but pain to the patient and injury to his prospects in the resettings and redressings that are so often indulged in with impunity prior to the "ninth day."

It is quite clear to me that the results in any case where this course is pursued might be improved by the surgeon dressing his patient *immediately* in an appliance that will be retained until union has occurred. This should, if possible, be done where the accident has occurred, in order to aid in his transportation.

Every physician and surgeon should select the method of dressing he prefers, and keep the material on hand ready for an emergency.

Failing to be thus prepared he has no alternative but to improvise an apparatus that will usually require replacement on the following day by one more perfect and adapted to the case if he succeeds in getting such constructed. This involves loss of time and untold vexations to the practitioner, and severe suffering to the patient, which, ordinarily might have been avoided and even then all concerned may feel happy if no further removals and redressings are necessary, especially if the injury be a severe one; one that needs absolute quietude and comfort.

Whatever may be the practice in Hospitals and by the more accomplished surgeons, many practitioners will discover in the preceding a hint at their own experience, recalling vividly their perplexity and anxiety during the treatment of such cases, the bandages that were too tight and must be loosened, too loose and must be tightened, splints that must be removed and repadded, a perineal band that excoriated and extension that ligated or blistered or both, the heel that became a source of unrelievable torture, and at last sloughing, became nearly as bad as the original difficulty; the heat of the limb from packing, the disagreeable condition arising from fluid applications or prevalent discharges, amid the bandages and pads of cotton or bran, all giving rise to discomfort to the patient, retarding recovery, increasing the chances of a bad result, and even imperiling life.

*The bed* on which the patient is to lie should be of *sufficient length,* have a firm bottom, without cords or springs, and be covered with a hard mattrass. No other should be used. If this cannot be obtained make a bunk of boards and scantling, with a board bottom and cover, with a mattrass or its equivalent, so that the body and fractured leg shall lie on a uniform plane. *A bad bed* has made many a *bad leg* despite the best appliances and otherwise most accomplished and careful surgery.

Before applying any dressing the surgeon should ascertain clearly the nature of the injury. Avoidance of inflicting pain is a cardinal principle, but it should never prevent the surgeon from ascertaining the exact character of the fracture. This is essential as a matter of treatment, and may become in any case a question of great importance in a court of law.

The method by which the fractured extremities are to be kept in apposition is one on which there would necessarily be a diversity of opinions, based on either theory or practice, or both. It is true that with all methods good results, in a proportion of cases, have been produced, the general impression with the profession being still that there is something wanting; that, as a whole, our appliances do not perfectly fill the demands.

The double inclined plane is almost entirely discarded because of its manifest defects; the method of Buck, while correct in its principle of extension, is altogether too unreliable for general practice, and Plaster-paris, for many reasons, can never become a favorite method with the general practitioner.

The defects of the most popular methods have been such that a high authority recommends their abandonment and reliance on extemporized apparatus. The dressing thus produced on the spot and which have given the most satisfaction, in fracture of the thigh, are usually, with slight modifications, the long straight splint of Desault. Though somewhat crude, its principle has met with general approval. Having during my army experience used this splint, by modifying it, so as to secure the fractured limb in position by suspension, I some time since undertook to supply myself with it, thus modified, for an emergency. It also occurred to me to add to it some means of *continuous and controlable extension.* Having thus begun, I sought to add one after another method of meeting pressing practical wants, until I have produced an apparatus that in every form of fracture of the lower extremity has given to myself and professional friends the greatest possible satisfaction, and which I have ventured to designate as the

HAMMOCK SPLINT.

The various practical adaptations possessed by the apparatus, will appear in the following brief description:

This instrument, without the Hammock Cloth, is exhibited in the accompanying cuts, in which the long outer and short inner splint is substituted by two rods, with a pad on the upper end of the larger. These rods are kept in position by cross rods and uprights, the whole being fastened at all points by clamps and screws, in such manner that the frame-work may be made high or low, wide or narrow, short or long, as desired to suit any size or length of limb, and adapt it to any variation in its size during treatment,—

*without the removal of any dressing or in the least disturbing the fracture.* In the figure the splint is arranged for the left leg but by turning the long rods, end for end, it is arranged for the right leg, and it is so constructed that these rods separate at a ferule in the middle in such manner that they may be so combined as to be adaptable to a leg of any length.

Fig. 2.

Figure 2 is an enlarged view of the boot extremity. The rods E, E, are supported by means entirely separate from the rods A. On these rods E E slides a slotted crossbar better seen in Fig. "1," and to which the rod that supports the foot-piece is attached by a clamp. Coiled wire springs surrounds the rods E E, the power of which, as is easily seen, may be so used as to force the cross-bar and its foot-piece toward the end of the instrument. This spring power is also controlled bar L, by which it may be increased or lessened at will. The crossbar being pushed against the spring and the extension fastenings made secure to it, we have a *continuous and controlable* means of extension. The foot being made fast to the foot-piece, the whole may be rotated laterally to any desired point and then secured by the screw in the crossbar, thus giving entire *command* of the *position of the foot and alignments of the limb.*

To the hooks noticed on the splint rods is attached the Hammock Cloth. This consists of good unbleached muslin or drilling, and extends from the heel end to the upper end of the body pad and is in the same maner fastened to the short rod, leaving between them enough cloth to form the "Hammock."

Having been attached to the long rod near its edge it is now cut along the short rod to its perineal end, where a slight sweep inward removes enough to leave the anus uncovered, and then cutting straight out forms the Hammock Cloth in an L shape, the short end of which goes around the body and fastens to the long rod, holding the body and splint securely together. Fig. 3 shows the dressing applied, H being the short leg of the Hammock Cloth, starting from and returning to the upper end of the long rod. It will be observed that the leg and thigh nates and back are all supported on one piece of cloth that should be without a wrinkle, and that, as a matter of mechanics, *the nates must afford the real basis of counter extension.* The perineal band is applied only as a means of security and should never be made tight enough to produce discomfort.

Fig. 3.

The extension is made by any means the surgeon may desire, adhesive plaster being undoubtedly the best. They should be so attached to a piece of tape as to furnish two tails on either sides to tie to the ends of the cross-bar.

I have found that these straps when constructed, as shown in Fig 4 to answer in a superior manner. B, B is a firm piece of bandage or tape, and A A two strips of adhesive plaster. Maws is the best cut, and applied around the roller as shown. Of these a pair is required, one being placed on either side of the leg. The adhesive plaster wraps around in a spiral manner, each side clasping the other. That *without strangulation* they hold very firmly, and *no bandage* is required, leaving *leg, ankle and foot entirely free from bandages* or any cover whatever. The tails are tied around either end of the cross-bar and may be long enough so that in case of fracture of the thigh the adhesive plaster need not come down near to the malleoli. When it is desired to produce counter extension, as in fracture of the leg, the plasters, constructed in the same manner, may be reversed and the tails tied to

Fig. 4.

the upper end of the splint. The upright seen in Fig. 1 is a moveable, irrigating apparatus that may be attached wherever desired, and the fluid applied from a cup by a cotton wicking syphon.

This apparatus when used in dressing fracture of the leg, can be adjusted to any length desired by the surgeon, and counter extension and extension effectually applied.

It is packed in a small box, only twenty-seven inches long, with which the physician may feel sure that in half an hour he can adjust any fracture of the leg and thigh, leaving his patient not only *comfortable* but *secure*.

This dressing, applied at once, is *permanent* and should not be removed until *union of bone* has occurred, nor the *ends of the bone moved in redressing*.

There being no bandaging and the whole limb in full view, with a foot-piece entirely under control, *if the surgeon can sight a gun, he ought to make a straight leg*.

The leg is suspended in a single layer of cloth beneath it, and is, therefore, *cool* as it possibly can be made, and affords all the facilities for irrigation or any other desired medication. Its convenience in *compound injuries* and *abscesses* is all that could be desired. *No excoriation or sloughing of the heel* can occur, *nor is the reparative material disturbed* at the after dressings, while the *extension is continuous and controllable* and counter extension obtained without discomfort from the nates.

Ample facilities for using the bed-pan are afforded without disturbing the fracture or erecting a trap through the mattrass.

But whatever dressing may be selected, there are several points to be considered, and among which is coolness, cleanliness, means of rectifying defects of position, and *absolute quietude of the fractured extremities*.

## Figures and Tables

**Figure f1:**
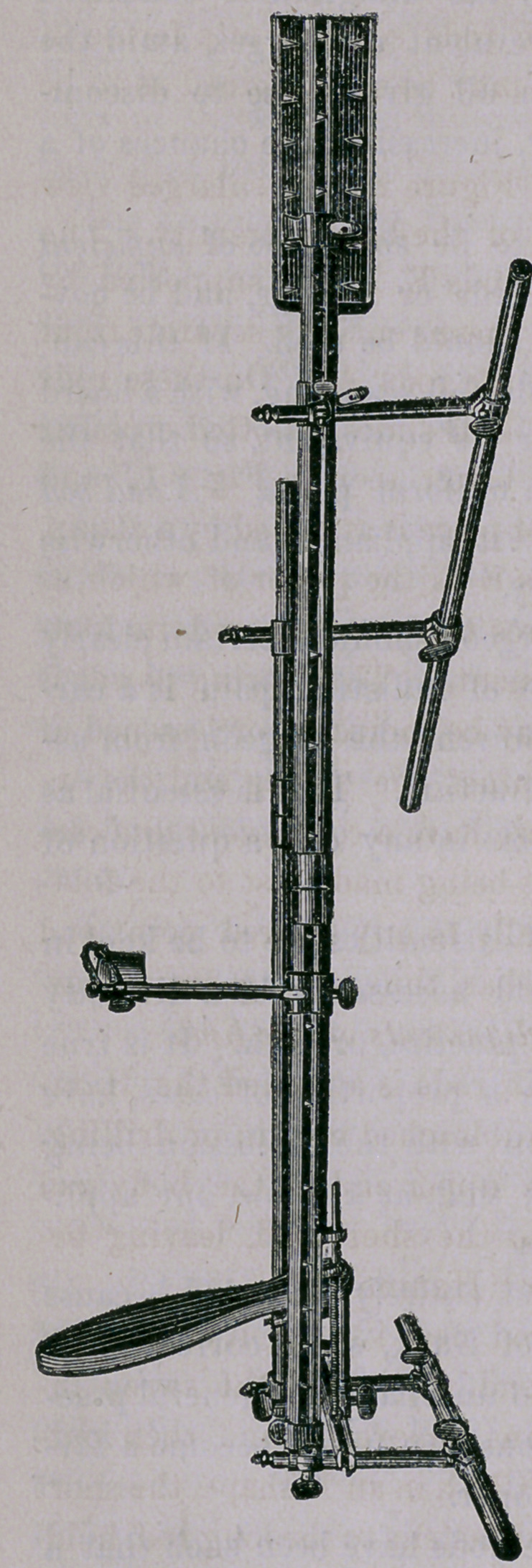


**Fig. 2. f2:**
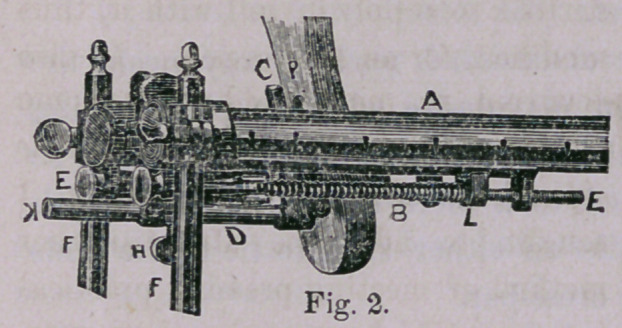


**Fig. 3. f3:**
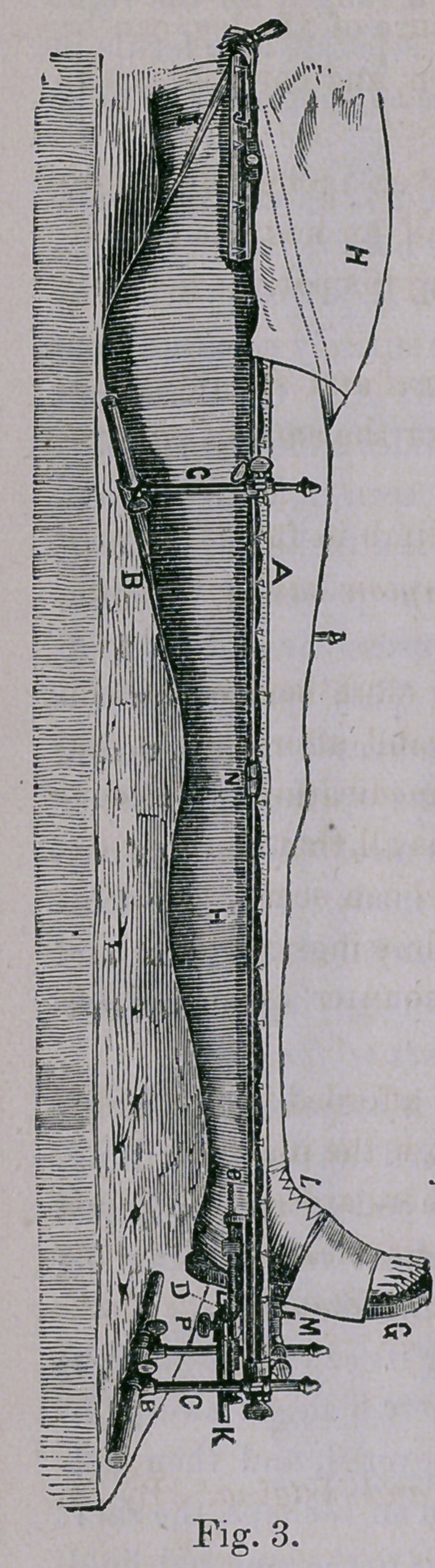


**Fig. 4. f4:**